# Using Explainable Machine Learning to Identify Determinants of Spinal Deformities in Children: It’s Not Only About What, but Also About How

**DOI:** 10.3390/healthcare14121601

**Published:** 2026-06-06

**Authors:** Dragica Bukumirić, Aleksandra Ilić, Mirjana Pajčin, Aleksandar Ćorac, Saša Milićević, Verica Jovanović, Živko Bojović, Ilija Doknić, Sindi Mitrović, Zoran Bukumirić, Zorica Terzić-Šupić, Jovana Todorović, Srđan Mašić

**Affiliations:** 1 Institute of Public Health of Serbia “Dr Milan Jovanovic Batut”, 11000 Belgrade, Serbia; 2 Department of Public Health, Medical Faculty, University of Pristina, 38220 Kosovska Mitrovica, Serbia; 3 Department of Surgery, Medical Faculty, University of Pristina, 38220 Kosovska Mitrovica, Serbia; 4 Faculty of Informatics and Computing, Singidunum University, 11010 Belgrade, Serbia; 5 Faculty of Organizational Sciences, University of Belgrade, 11010 Belgrade, Serbia; 6 Department of Physical Medicine and Rehabilitation, Faculty of Medicine, University of Belgrade, 11000 Belgrade, Serbia; sindizmitrovic@gmail.com; 7 Institute for Medical Statistics and Informatics, Faculty of Medicine, University of Belgrade, 11000 Belgrade, Serbia; zoran.bukumiric@med.bg.ac.rs; 8 Department of Clinical Research, Faculty of Health Sciences, University of Southern Denmark, 5230 Odense, Denmark; 9 Institute for Social Medicine, Faculty of Medicine, University of Belgrade, 11000 Belgrade, Serbia; 10 Department of Primary Health Care and Public Health, Faculty of Medicine, University of East Sarajevo, 73300 Foca, Bosnia and Herzegovina

**Keywords:** spinal deformities, children, explainable machine learning

## Abstract

Background: Spinal deformities in children represent a relevant public health issue, with possible long-term consequences. Timely identification of their determinants is essential for adequate prevention. Methods: This study was a secondary analysis of data from the 2019 Serbian National Health Survey, including 1309 children aged 5–14 years. Logistic regression with LASSO regularization and multiple ML algorithms were tested, with XGBoost selected as the optimal model. Class imbalance was addressed using class weighting and SMOTE. Model interpretability was achieved using SHAP analysis. Results: The prevalence of spinal deformities was 8.6%. Univariable analyses showed that age, poorer self-rated health, chronic illness, recent injuries, and pes planus were significantly associated with spinal deformities. Family-related variables showed no significant associations. Among the evaluated models, XGBoost demonstrated the most stable performance across the applied evaluation metrics and the best balance between predictive performance and interpretability. SHapley Additive exPlanations (SHAP) analysis showed that pes planus was the strongest determinant, followed by age and chronic illness, while socio-demographic and family factors had minimal influence. Conclusion: Explainable machine learning models, particularly XGBoost combined with SHAP, can allow for the identification and interpretation of key determinants of spinal deformities in children. Pes planus was shown to be modifiable and relevant associated determinant, supporting its importance in early screening and preventive strategies.

## 1. Introduction

Spinal deformities in children represent changes in the normal position or shape of the spinal column, which can affect the proper growth, development, function, and quality of life of the child. They most often appear in childhood during the period of the most intensive growth and development, slowly and imperceptibly. The most prevalent ones are scoliosis, kyphosis, and lordosis [1,2,3]. Spinal deformities can occur due to genetic predispositions, developmental disorders, poor posture, insufficient physical activity, neuromuscular diseases, excess weight, or other factors [1,2,3,4]. If deformities remain undetected or are not treated, they are not only an aesthetic problem, but can lead to serious functional disorders, including chronic pain, movement restriction, and even problems with breathing and cardiac issues in severe deformities. All these issues can significantly limit the child’s ability to function in daily life [5,6]. Deformities can affect self-confidence and social interactions, further complicating their development, reducing the quality of life, lowering school performance, and increasing the likelihood of difficulties in socialization [5,6,7]. Spinal deformities in children are associated with important functional, developmental, and quality-of-life consequences and they could have negative influence on mental health and social inclusion [2,8,9]. Inadequately or untimely treated spinal deformities can get progressively worse and be associated with the need for complex surgical interventions that are invasive and require long-term rehabilitation [10].

Early identification of spinal deformities may support timely preventive and corrective interventions, along with support for families, through schools, health, and social institutions [10]. Identification of spinal deformities on a large scale can be achieved through the organized, systematic examinations of all school-aged children. This allows for timely intervention on one hand, but also provides time and space to conduct educational sessions for children, parents and teaching staff on the importance of proper posture and regular physical activities [11]. If detected early, physiotherapy and orthopedic aids could significantly reduce the progression of deformity and reduce the overall burden on the healthcare system [12]. However, despite the importance of early detection, there is limited evidence on broader population-level determinants of spinal deformities in children, particularly using modern explainable machine learning approaches that could support earlier identification of children at increased risk.

Overall prevalence of idiopathic scoliosis in adolescents is 0.47–5.2% [13]. A study conducted in Jakarta found that school children (from grades 3 to 6) had a scoliosis prevalence of about 7%, with a higher prevalence in girls who were significantly taller and had lower BMI values [14]. During the 2013 National Health Survey of Serbia, 4% of examined children had spinal deformities, whereas the 2019 study found deformities in 8.7% of children [15,16]. Although differences in prevalence between the two national surveys may partly reflect differences in reporting, awareness, or detection practices, the observed increase highlights the need for improved understanding of determinants associated with spinal deformities and for approaches that may support earlier identification of children at increased risk.

So far, studies have tried to explain the contribution of various factors to the development of spinal deformities in children, using classic statistical methods in data analysis [1]. Today, newer techniques such as machine learning (ML) are increasingly being introduced. Machine learning is a set of algorithms that enable computers to learn from data and make predictions or decisions without being explicitly programmed. In pediatric healthcare, ML is used for diagnostic support, prognostic modeling, and therapeutic planning [17]. In Pediatric Orthopedics, machine learning models showed over 90% accuracy in classification and curve prediction [18].

The study by Lv and associates [19] applied ML models to predict Adolescent idiopathic scoliosis (AIS) in the children and adolescent population. The authors showed that the artificial neural network model (ANNM) has good prediction and can help clinicians with the diagnosis and treatment of AIS. A systematic review of machine learning for predicting progression of adolescent idiopathic scoliosis found that although more accurate than traditional methods, these models have limited clinical application due to their complexity and insufficient interpretability [20]. Greater interpretability of machine learning models means easier understanding and enables healthcare experts to make decisions that lead to higher quality healthcare services [21].

To the best of our knowledge, no published studies have examined the application of explainable machine learning models to identify determinants of spinal deformity in children. Unlike traditional statistical approaches, explainable machine learning models may capture complex nonlinear relationships and interactions between determinants while simultaneously enabling interpretation of their relative contribution to the outcome. Therefore, the aim of our study is to identify the determinants associated with spinal deformities in children using explainable machine learning approaches and explain their relative contribution to the outcome so that timely preventive actions can be planned.

## 2. Materials and Methods

This study presents a secondary analysis of data from the cross-sectional 2019 Serbian National Health Survey, focusing on children aged 5 to 14. The sample was constructed using multi-stage, stratified, and cluster sampling in accordance with the methodological standards of the European Health Interview Survey (EHIS) [16,22].

In the present study, spinal deformities were analyzed as a composite outcome variable including any reported spinal deformity identified during routine systematic health examinations in school-aged children. The available national survey data did not differentiate specific deformity subtypes such as scoliosis, kyphosis, or lordosis.

### 2.1. Research Sample

The research was conducted on a nationally representative sample of Serbian residents, assessed on the basis of the population census conducted in 2011.

### 2.2. Target Population

The present secondary analysis focused exclusively on children aged 5–14 years included in the 2019 Serbian National Health Survey.

### 2.3. Sample Type

The primary research used a stratified two-stage sample of groups. A random sample of census circles (groups of households) was selected with probability proportional to the size in the first stage. A sample of households in each census round was selected with equal probability in the second stage.

### 2.4. Measurement Instruments and Variables

The measurement instruments were constructed in accordance with the questionnaire of the European Health Interview Survey (EHIS) and adapted to the specifics of our area [22].

Three types of questionnaires and a measurement form were used:

1. Household information panel, used to collect information on household members and socio-economic characteristics of the household (household-reported data).
2. Questionnaire for household members aged 5 years and older. Two versions were used: one for adults aged ≥15 years (self-reported data) and another for children aged 5–14 years, completed by parents or guardians (parent-reported data).
3. Self-administered questionnaire, which included sensitive questions completed independently by household members aged ≥15 years (self-reported data).
4. Measurement form for objective findings household members aged ≥15 years.

For secondary analysis of children’s health disorders as outcomes of interest in the domain of functional limitations and weight gain, the following data were taken from the electronic database of the Institute of Public Health of Serbia:

The analysis included the following characteristics of children:

Outcome variable: Presence of spinal deformity (yes/no), identified during routine systematic health examinations and reported by the parent in the National Health Survey.

Health determinants:

- Demographic and socio-economic characteristics of respondents: gender (male/female), age (in years) and type of household settlement (village/city).
- Children’s health status (assessment by parents): overall health of children (very bad, bad, average, good, very good), existence of functional impairments (any spinal deformity, pes planus), existence of chronic disease (yes/no), existence of injuries/accidents in the last 12 months (yes/no)
- Value of body height and body weight, nutritional status of children (BMI classification into underweight, normal weight, overweight, and obesity categories according to WHO criteria) [23]
- Physical activity and sports activities of children (parent-reported number of days per week in which the child participated in sport or fitness activities).

Family characteristics included: number of household members; household income quintiles (categorized as poorest, second, middle, fourth, and richest according to the national survey methodology); average age of other family members; proportion of children living with both parents/guardians versus one parent/guardian; average educational level of other family members based on the highest completed level of education (no formal/incomplete primary education, primary school, secondary school, vocational college/college, university degree, and postgraduate education including master’s degree, magisterium, or PhD); average number of days per week with sport or fitness activities among other family members; and average self-rated health of other family members. Family-level variables derived from individual household members (e.g., age, education, physical activity, and self-rated health) were calculated as arithmetic means or proportions within each household.

### 2.5. Data Analysis

Data analysis included classical statistical methods and Explainable Machine Learning models.

Statistical analysis: We calculated descriptive statistics based on the type of data. For continuous variables, we reported the mean and standard deviation if the data followed a normal distribution. If the data did not meet this normality, we used the median (range). Categorical variables are presented as absolute numbers and percentages (n, %). Ordinal variables were encoded according to their natural order, whereas nominal categorical variables were one-hot encoded before analysis. To understand what causes spinal deformities, we used a two-step analysis. First, we used simple logistic regression to find possible determinants. Variables that had a *p*-value less than 0.1 were included in the next step, the multivariable model. This way, we avoided leaving out important factors too early in the process. All candidate determinants were entered into the LASSO model. LASSO regularization was then used to identify the most relevant determinants while reducing model complexity and overfitting. Ten-fold cross-validation identified the optimal regularization parameter (λ). Only determinants with non-zero coefficients remained in the final model. Multicollinearity was assessed using variance inflation factors (all VIFs < 1.5), residual diagnostics showed no substantial deviations, and linearity of continuous predictors was confirmed using the Box–Tidwell procedure. We evaluated model performance using goodness-of-fit and ROC analysis. Statistical significance was set at *p* < 0.05. All analyses were performed using IBM SPSS Statistics for Windows, version 31.0 (IBM Corporation, Armonk, NY, USA), Python version 3.11 (Python Software Foundation, Wilmington, DE, USA) or the R software environment version 4.5 (R Core Team, 2025).

**Figure 1:**
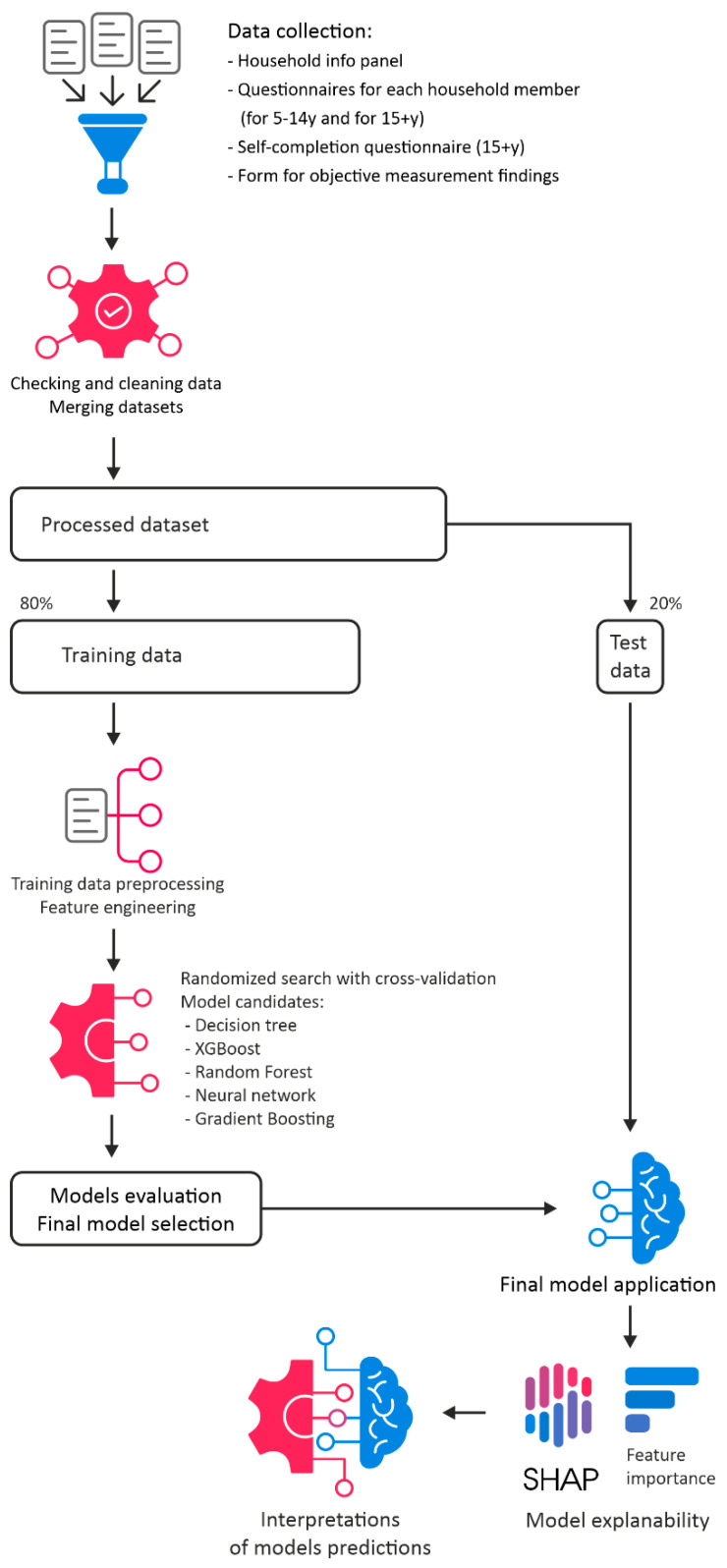
Workflow of the machine learning analysis process, from data collection and preprocessing to model training, evaluation, and explainability.

Explainable Machine Learning: Given that the aim of the research is to identify determinants and assess their relative impact on spinal deformity, various machine learning models capable of capturing non-linear relationships and interactions between variables were applied: The following machine learning algorithms were evaluated: Decision Tree, XGBoost, Random Forest, Neural Networks, and Gradient Boosting. These algorithms were selected because they are widely used for tabular biomedical classification tasks, represent complementary modelling approaches, and demonstrated promising performance during the preliminary model training. Before any feature selection, preprocessing related to class imbalance, hyperparameter tuning, or model training, the dataset was divided into training and testing subsets. A stratified 80:20 split was used in order to preserve the proportion of positive spinal deformity cases in both subsets. The test set was kept completely untouched throughout model development and was used only once, for the final evaluation of model performance (Figure 1).

LASSO regression was performed using the training data to select relevant variables and reduce overfitting. The stability of determinant selection was additionally assessed using a stability selection approach, which involved repeated sampling of the training data and monitoring the frequency of selection of individual variables in LASSO models. Variables consistently selected in ≥70% of iterations were treated as robust determinants. Since the outcome of interest (spinal deformity) was present in 8.6% of subjects, special attention was paid to the treatment of unbalanced classes. By applying class weighting in the loss functions and the SMOTE algorithm for synthetic expansion of the minority class in the training set, we aimed to improve the models’ ability to recognize and classify positive cases.

Hyperparameter tuning was done using the RandomizedSearchCV function from the scikit-learn library. To use it, it is necessary to determine an interval for each hyperparameter of interest, from which training values will be randomly selected. The search spaces were selected heuristically using commonly applied ranges for small-to-moderate tabular datasets. The number of iterations was fixed, and a random seed was set to improve reproducibility. The advantage of this approach compared to GridSearchCV is that it allows the number of training iterations to be explicitly defined and the less important hyperparameters will not, as a rule, get the same resources as the more important parameters.

Model performance was evaluated using metrics adjusted for unbalanced categorical outcomes: Area Under ROC Curve (AUC), Matthews Correlation Coefficient (MCC), Sensitivity (Sn, Recall), Specificity (Sp), PPV (Positive Predictive Value, Precision), Accuracy, and F1-score for the positive class. Metrics of applied models are presented in Table 1. These models were obtained with the application of class weighing in the cost function but without SMOTE preprocessing since it did not enhance performance during the model development.

XGBoost was selected as the most acceptable model, which was the most stable based on the applied metrics, and at the same time, in direct comparisons of the applied models, it proved to be the best compromise between performance and interpretability. For the XGBoost model, an analysis was conducted on the test data to assess the importance of individual determinants using the SHAP (SHapley Additive exPlanations) method [24]. SHAP values enabled the quantification of the contribution of each independent variable to the prediction and can show the impact both at the set level and at the individual prediction level. Global SHAP analysis was used to assess the overall relative contribution of determinants, while local SHAP waterfall plots were used to illustrate the contribution of individual variables in representative predictions.

## 3. Results

The 2019 Serbian National Health Survey included 13,178 respondents from 5514 families. In 997 families with 4728 members, there were 1454 children aged 5–14. A total of 1309 children had data on the presence or absence of spinal deformity, and they were included in the analysis with their families. The prevalence of spinal deformities was 8.6% (112/1309 children).

Table 2 shows the characteristics of the study participants. For each observed category, corresponding numerical indicators are presented.

In univariable analyses, of the variables that describe children’s characteristics, spinal deformities were significantly more common in older children (*p* = 0.028), worse overall health of children (*p* < 0.001), children with chronic illness (*p* < 0.001) and injuries in the past 12 months (*p* = 0.020) and children with pes planus (*p* < 0.001) (Table 2).

Data related to other family members are presented as summary data on the rest of the family and are obtained as the arithmetic mean of numerical and ordinal variables or proportions of the selected category in nominal ones. The median number of family members was 5, range 2–16. The families had approximately the same income categories. Equal representation of male and female family members, the average age was 44.1 years, family members most often had a secondary level of education, the family most often rated its health as good, and about 10% of children did not live with both parents/guardians. Characteristics of the families in which children live are presented in Table 3.

In univariable analyses, none of the variables used in the analysis, which describe the family, were statistically significant in relation to the presence of spinal deformities in children (*p* > 0.05).

**Figure 2:**
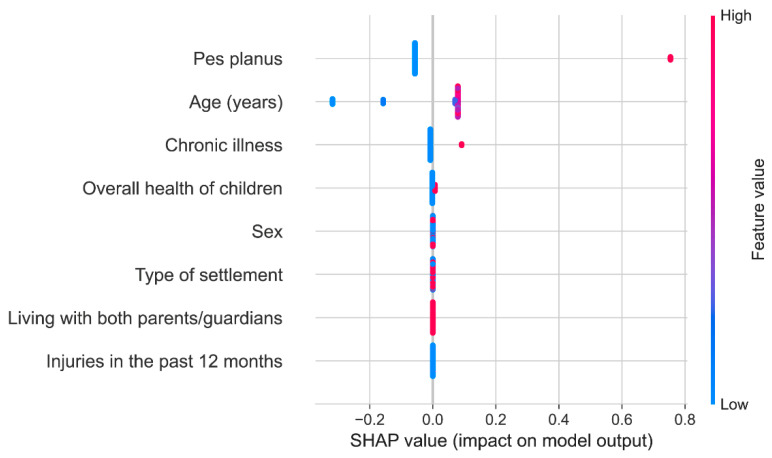
SHAP summary plot shows the relative importance of factors in the XGBoost model for predicting spinal deformity in children.

The SHAP summary plot presented in Figure 2 describes how different factors contribute to the XGBoost model’s prediction of spinal deformities in children. The pes planus shows the strongest association with an increased probability of spinal deformities, reflected in the higher (pink) SHAP values. Older children generally show higher SHAP values, indicating an increase in probability with age. Having a chronic illness or generally poor health increases the probability of having spinal issues. Factors like sex, settlement type, family structure, and recent injuries show minimal influence on the model’s performance.

**Figure 3:**
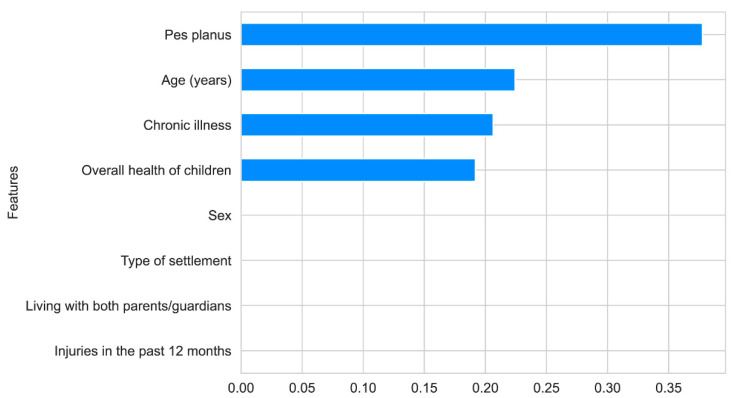
Feature importance of the final XGBoost model for predicting spinal deformities in children.

Feature importance of the final model (Figure 3) shows the order and relative importance of the factors that contributed the most to the model in determining the probability of spinal deformity in children. The analysis points out that pes planus are the most important factor, with the highest importance value, meaning they strongly increase the probability of spinal problems. Other factors, such as age, chronic illness, and number of children in the family, have moderate effects but are less influential than pes planus.

**Figure 4:**
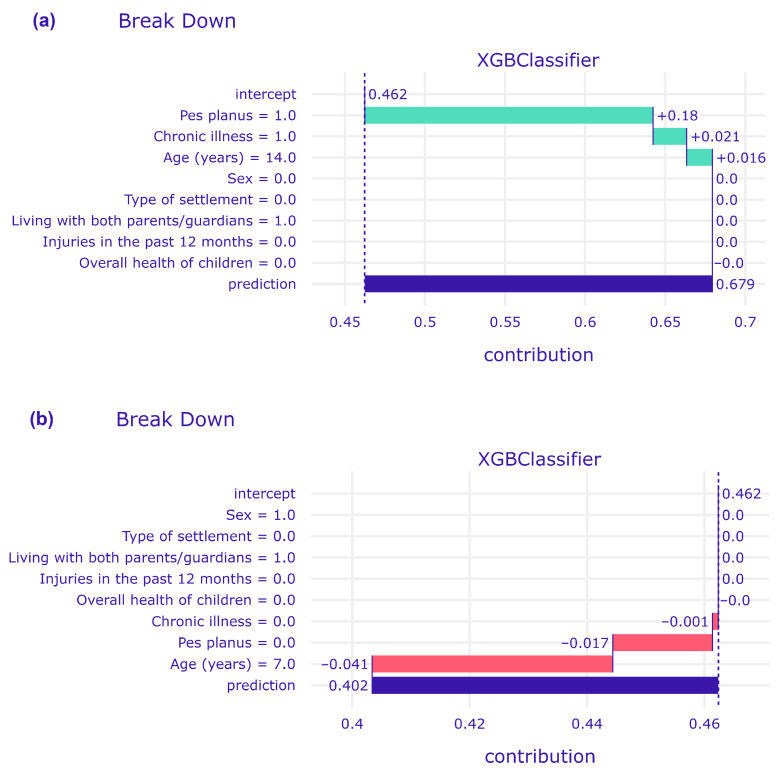
SHAP waterfall breakdown plots for individual predictions of spinal deformity. (**a**) a positive prediction (presence of spinal deformity), (**b**) a negative prediction (absence of spinal deformity).

The SHAP breakdown waterfall plot (Figure 4) shows how individual features contributed to the predicted probability of spinal deformity for two representative cases. First case (Figure 4a), the model yields a final predicted probability of spinal deformity of 0.657. The presence of pes planus represents the strongest positive factor, accounting for the largest upward shift from the baseline prediction, followed by chronic illness and age. Other variables have negligible contributions. Second case (Figure 4b), the predicted probability is substantially lower (0.462). Younger age and the absence of pes planus contribute negatively to the prediction. All remaining features show minimal influence on the final outcome.

These results highlight that anatomical and developmental characteristics (particularly Pes planus) are the primary determinants of the model’s prediction. In contrast, social, environmental, and demographic variables exert limited influence on the estimated probability for this specific individual.

## 4. Discussion

The aim of this study was to identify the determinants of spinal deformities in children with the application of modern methods using artificial intelligence, and the identification of the most important determinants. Modern methods were used to reveal non-linear relationships between potential determinants and outcomes that are difficult to detect with classical statistical methods (in our case, logistic regression). Most of the studies so far were focused on diagnoses of deformities such as scoliosis, kyphosis, and lordosis [1,2,25]. Our focus was on the identification of determinants that can influence the existence of any type of spinal deformity in children in order to react in a timely manner and enable detection at the earliest possible stage.

In the Serbian healthcare system, systematic examinations of children, conducted every two years, enable early detection of spine changes and to prompt intervention to prevent further progression and ensure correction. Evidence-based knowledge gives us the opportunity to monitor and prevent certain conditions with timely interventions.

The comparison indicated that traditional models, such as logistic regression, achieved good overall accuracy (AUC = 0.752). However, their sensitivity was very low (0.027), indicating difficulty in identifying children with spinal deformities. In contrast, AI-based models (particularly XGBoost) demonstrated more balanced performance across metrics: sensitivity (0.375), specificity (0.931), and MCC (0.259). This balance suggests a stronger ability to distinguish between positive and negative cases. The MCC metric proved most reliable because it incorporates all components of the confusion matrix and performs well with unbalanced datasets. Although sensitivity remained low, due to the small number of positive cases, XGBoost still achieved higher MCC and F1-scores, confirming its greater accuracy and stability. The use of class weighting and SMOTE further improved the detection of positive cases with minimal loss in overall accuracy. Considering both performance and interpretability, XGBoost combined with SHAP analysis appears to be the most appropriate approach for the early identification of spinal deformity risk in children. Integrating such models into health information systems and school screening programs could enhance personalized prevention strategies in pediatric orthopedics.

In our sample, it was shown that the most significant determinants of the presence of spinal deformity in children aged 5 to 14 years are the presence of pes planus, older age, the existence of chronic diseases and injuries within 12 months.

The first and most important determinant of the presence of spinal deformity in children in our study is pes planus. As a deformation of the foot in which it is flattened due to the reduction or loss of the medial longitudinal arch, pes planus certainly contributes to changes in the posture of the pelvis and spinal column [26]. From a biomechanical perspective, pes planus involves excessive pronation of the foot, leading to altered lower-limb alignment, including internal rotation of the tibia and femur and anterior pelvic tilt [27]. These biomechanical changes may induce compensatory postural adaptations along the spine, which are primarily associated with functional or postural deviations and, over time in children, may contribute to the development of structural spinal deformities. Long-term changes in body posture have been shown to significantly affect spinal curvature [28], which may help explain the higher prevalence of spinal deformities observed in older children. Furthermore, a significant statistical relationship between the degree of pes planus and posture-related parameters has been reported, with higher degrees of pes planus correlating with reduced flexibility and weaker isometric trunk strength, contributing to alterations in the lumbar curve and overall posture, that is, postural disorders [26]. The importance of pes planus as a determinant of spinal deformity, and even spinal degenerative joint disease later in life, has also been previously documented [29]. Consistently, the study by Vlad et al. [30] identified an association between flatfoot and scoliosis in children aged 8–12 years, highlighting the importance of early identification and the implementation of targeted preventive programs addressing postural deficiencies in children.

Age was positively associated with the presence of spinal deformity in our sample, which is consistent with previous findings and the physiological characteristics of the preadolescent period, in which growth spurts are known risk factors. As children age, the proportion of children with spinal deformity increases. Today, children are also exposed to a number of environmental factors, including the high prevalence of the use of mobile phones and other electronic devices, increased sitting time at school and at home, low levels of physical activity [28]. During the school period, children are exposed to carrying heavy backpacks with books, which often asymmetrically loads the spine [31]. Poor posture in younger children for a long period of time inevitably leads to postural and structural changes in the spine, which become more pronounced and visible with age. The rapid growth that is characteristic of this period can lead to an imbalance of bone development and muscle tone, which also contributes to the development of spinal deformities (especially scoliosis) and, if not recognized on time, leads to deformity that worsens during growth [32,33].

Identification of pes planus and age as the most important determinants is of great importance in countries that have organized screening in preschool age, because flexible pes planus in children is a variable risk factor and a condition that can be corrected [29]. By reacting quickly and applying adequate measures, the occurrence of spinal deformities can be prevented.

Poor body posture combined with pes planus certainly warns us of a more detailed and comprehensive approach and monitoring of such children in terms of timely action and prevention of the development of deformities or prevention of more severe forms at a later age.

Sex was not a significant determinant of spinal deformity in our study, although it has been shown in several earlier studies that there are differences in the prevalence of scoliosis, kyphosis and kypholordosis between boys and girls. In previous studies, it was determined that scoliosis is more common in girls, while kyphosis and lordosis were more common in boys [28]. Since we analyzed all spinal deformities together, this may be the reason why sex was not detected as a significant statistical determinant. One systematic review showed a prevalence of scoliosis of 3.1%, and this frequency varied depending on sex, severity of scoliosis, and between idiopathic and congenital scoliosis [34]. A study involving 697,043 primary and secondary school students in mainland China revealed a prevalence of scoliosis of 1.02%, with a higher incidence in girls (1.54 times higher) [35]. The prevalence of scoliosis was 2.52%, with a higher incidence in girls (3.11%) compared to boys (1.96%) [36].

Previous studies on spinal deformities in children have mostly focused on prevalence, clinical examination, or prediction of scoliosis progression [19,20,25,31]. For example, Petrovic et al. reported a prevalence of spinal deformities of 23.1% among children aged 7–11 years, with kyphosis, scoliosis, and hyperlordosis analyzed separately, while sex, age, and BMI were not identified as significant predictors [25]. In contrast, the present study used a nationally representative sample and an explainable machine learning approach to identify broader population-level determinants of any spinal deformity. Our finding that pes planus was the strongest determinant is consistent with studies suggesting that foot and spinal deformities may coexist in children due to biomechanical interactions between foot posture, lower-limb alignment, and spinal loading [26,29,30]. Furthermore, unlike previous machine learning studies that focused on predicting scoliosis severity or progression using clinical and radiographic data [19,20], our study used survey-based health and lifestyle variables together with SHAP analysis to identify interpretable determinants relevant for population-level screening and prevention. The presence of chronic diseases in children was singled out as a significant determinant of spinal deformity. Based on the pre-structured questionnaire, there is no information about the cause of chronic diseases, but for certain chronic diseases there is evidence that they are related to the occurrence of spinal deformities in children, such as respiratory diseases [37,38]. There are not many studies that describe the importance and impact of chronic diseases on the development of spinal deformities in children, mostly the studies are based on the impact of spinal deformity on chronic disease, most often the impact of scoliosis on diseases of the respiratory system and neuromuscular diseases [39]. Diseases associated with hyposthenic body posture, reduced muscle tone, reduced physical activity and a sedentary lifestyle can significantly contribute to the development of spinal deformity, especially during the period of rapid development [37].

Injury in the last twelve months was also significantly associated with spinal deformities in our study. There is no possible explanation for this finding, but based on the existing knowledge, it can be concluded that certain injuries that are associated with longer periods of limited movement and with physical inactivity, as well as a forced position for a longer period of time, can affect changes in body posture and the development of spinal deformities. However, as this association may be bidirectional, future studies could investigate the presence of deformity before the injury, as well as whether the spinal deformity has an influence on the more frequent occurrence of the injury or whether the injury affected the spinal deformity. Individuals with spinal deformities exhibit pathological changes such as postural abnormalities, standing instability, and gait changes. Vertebral deformation and body asymmetry cause motor imbalance, which threatens balance and can also affect respiratory and cardiac functions [31].

Analyzed potential family-related determinants did not show any association with the presence of spinal deformity in children, although certain conditions in the family can contribute to the development of spinal deformity in children, such as nutritional factors and parental idiopathic scoliosis [31].

When comparing the performance of the classic logistic regression model with other exML models, it can be noted that the logistic regression classified the participants well overall (AUC 0.752), but the MCC, sensitivity, and F1 score for the positive class were lower than those of the exML model. While exML models with corrections for unbalanced classes showed better overall model explainability and relative influence of individual variables. Similar results were obtained in the research of Doknic et al. [40] in prediction of thrombosis in patients with acute myeloid leukemia. In addition, in the case of unbalanced classes, in the case of the rarer class, even one good or wrong classification changes the evaluation parameters to a higher degree than in the case of the dominant class.

In the pediatric population, where early intervention is crucial, such models can play a key role, not only in detecting risk, but also in shaping personalized and preventive strategies based on understanding the relationship between risk factors and expected outcomes.

### Study Limitations

The main strengths of this study are the use of a nationally representative sample and the application of modern machine learning algorithms. However, there are several important limitations that should be considered when interpreting the results. A complex sample design, using multi-stage, stratified, and cluster sampling, may lead to increased correlation between respondents within the same clusters (e.g., households or settlements), and the potential effects of the cluster structure could remain uncontrolled. The study relies on secondary data collected for the purpose of broader health research, so the number of available determinants was limited. Some important factors for the development of spinal deformity (e.g., genetic predisposition, quality of work environment at school, length of sitting, detailed orthopedic findings, type of chronic disease) were not included in the analysis, which may affect the completeness of the model. An additional limitation is that spinal deformities were analyzed as a single composite outcome variable. The available EHIS-based data and the questionnaire for children younger than 15 years included only information on the presence of spinal deformity, without specification of deformity subtypes such as scoliosis, kyphosis, or lordosis. Consequently, separate analyses for individual deformities were not feasible, and this approach may have attenuated subtype-specific associations, particularly sex-related differences. The low prevalence of the target outcome of 8.6% poses a challenge for model training and may lead to low sensitivity and poorer performance of the classifier. Although efforts were made to balance classes and evaluate models using metrics adjusted for unbalanced data, the risk of bias still cannot be completely excluded. Although SHAP algorithms were used in the research to analyze the contribution of determinants, the result should be interpreted as exploratory and not as proof of causality. In order to draw conclusions about cause-and-effect relationships, further research with longitudinal data is necessary to determine the time sequence between determinants and the appearance of deformities.

## 5. Conclusions

The problem of spinal deformities in children deserves special attention and in-depth analysis. It is necessary to understand the factors that contribute to their occurrence, to recognize the symptoms and signs in time, as well as to apply the appropriate methods of prevention and treatment. Only in this way can we ensure that children grow and develop with as few negative consequences as possible for health, physical appearance and quality of life. Using new methods in the analysis of large data sets can help us identify new determinants as well as their mutual relationship.

## Figures and Tables

**Table 1:** Performance metrics of the evaluated machine learning mode.

Model	AUC	MCC	Sn	Sp	PPV	Accuracy	F1
Logistic Regression Classic	0.752	0.081	0.027	1.000	0.375	0.913	0.026
Decision Tree	0.755	0.229	0.563	0.821	0.170	0.805	0.261
XGBoost	0.774	0.259	0.375	0.931	0.261	0.897	0.308
Random Forest	0.756	0.296	0.563	0.878	0.231	0.805	0.327
Neural Networks	0.697	0.132	0.045	0.996	0.500	0.916	0.083
Gradient Boosting	0.749	0.243	0.063	1.000	1.000	0.943	0.117

**Table 2:** Characteristics of the children in the study.

Variables	Total n = 1309	With Spinal Deformity n = 112	No Spinal Deformities n = 1197	*p*-Value
Sex, n (%)				0.881
male	675 (51.6%)	57 (50.9%)	618 (51.6%)	
female	634 (48.4%)	55 (49.1%)	579 (48.4%)	
Age in years, n (%)				0.028
6	153 (11.7%)	3 (2.7%)	150 (12.5%)	
7	121 (9.2%)	6 (5.4%)	115 (9.6%)	
8	131 (10.0%)	13 (11.6%)	118 (9.9%)	
9	134 (10.2%)	13 (11.6%)	121 (10.1%)	
10	138 (10.5%)	15 (13.4%)	123 (10.3%)	
11	139 (10.6%)	16 (14.3%)	123 (10.3%)	
12	153 (11.7%)	18 (16.1%)	135 (11.3%)	
13	159 (12.1%)	12 (10.7%)	147 (12.3%)	
14	181 (13.8%)	16 (14.3%)	165 (13.8%)	
Type of settlement, n (%)				0.084
urban	704 (53.8%)	69 (61.6%)	635 (53.0%)	
other	605 (46.2%)	43 (38.4%)	562 (47.0%)	
Overall health of children, n (%)				<0.001
Very good	1045 (79.8%)	78 (69.6%)	967 (80.8%)	
Good	234 (17.9%)	27 (24.1%)	207 (17.3%)	
Average	24 (1.8%)	4 (3.6%)	20 (1.7%)	
Poor	6 (0.5%)	3 (2.7%)	3 (0.3%)	
Very poor	0 (0.0%)	0 (0.0%)	0 (0.0%)	
Chronic illness, n (%)	99 (7.6%)	21 (18.8%)	78 (6.5%)	<0.001
Injuries in the past 12 months, n (%)	75 (5.7%)	12 (10.7%)	63 (5.3%)	0.020
Leisure time sport or fitness (days per week) median (range)	3 (0–7) (mean 2.70)	2 (0–7) (mean 2.71)	3 (0–7) (mean 2.70)	0.989
Nutritional status, n (%)				
Normal weight (reference)	685 (60.7%)	54 (57.4%)	631 (61.0%)	-
Underweight	37 (3.3%)	3 (3.2%)	34 (3.3%)	0.961
Overweight	265 (23.5%)	25 (26.6%)	240 (23.2%)	0.438
Obese	141 (12.5%)	12 (12.8%)	129 (12.5%)	0.802
Pes planus, n (%)	148 (11.3%)	43 (38.4%)	105 (8.8%)	<0.001

**Table 3:** Characteristics of the families children live in.

Variables	Total n = 1309	With Spinal Deformity n = 112	No Spinal Deformities n = 1197	*p*-Value
Number of household members, median (range)	5 (2–16) (mean 5.378)	5 (2–16) (mean 5.402)	5 (2–16) (mean 5.376)	0.893
Income quintiles, n (%)				0.453
1—Poorest	300 (22.9%)	26 (23.2%)	274 (22.9%)	
2—Second	290 (22.2%)	25 (22.3%)	265 (22.1%)	
3—Third	267 (20.4%)	16 (14.3%)	251 (21.0%)	
4—Fourth	275 (21.0%)	25 (22.3%)	250 (20.9%)	
5—Richest	177 (13.5%)	20 (17.9%)	157 (13.1%)	
Proportion of females among other family members, median (range)	0.5 (0.0–100.0) (mean 0.527)	0.5 (0.0–100.0) (mean 0.537)	0.5 (0.0–100.0) (mean 0.526)	0.512
Average age of other family members, median (range)	44.1 (22.0–80.3)	44.9 (23.5–69.0)	44.0 (22.0–80.3)	0.300
Living with both parents/guardians, n (%)	1177 (89.9%)	95 (84.8%)	1082 (90.4%)	0.064
Average educational level of other family members, median (range)	3 (1–6) (mean 3.039)	3 (1–5.5) (mean 3.123)	3 (1–6) (mean 3.032)	0.281
Average self-rated health of other family members (1—very good to 5—very poor), median (range)	2 (1–5) (mean 2.045)	2 (1–3.5) (mean 2.108)	2 (1–5) (mean 2.040)	0.260

## Data Availability

The Serbian Health Survey, which the study analyzed, was conducted by the Institute of Public Health of Serbia. The data can be obtained through contact with the Institute of Public Health of Serbia via: email: kabinet@batut.org.rs.
